# Atlanta Society of Medicine

**Published:** 1890-04

**Authors:** 


### ATLANTA SOCIETY OF MEDICINE.

MARCH 18, 1890.

Atlanta Society of Medicine met, and in the absence of President and Vice-President, Dr. H. P. Cooper was called to the chair.

Dr. J. McF. Gaston reported a case of vesical urinary calculus. The patient was an old man of seventy-six years, under the care of Dr. J. S. Horsley, at West Point, Ga., who had resorted to crushing the stone and washing out the fragments some months ago with temporary relief. But finding that there were calculi located in a pouch at the posterior part of the bladder, shut off by an enormously large prostate, which could not be reached by the lithotrite, he concluded that a surpra-pubic cystotomy was indicated. Dr. Gaston was summoned by telegram to assist him on the 17th instant, and fully concurred in the propriety of undertaking this operation, not simply for the removal of the calculi, but for the subsequent drainage of the bladder to relieve the cystitis. Having the assistance of Drs. Winston and Pattillo, with the presence of Mr. John Pattillo, a student of medicine, everything was provided for the different steps of supra-pubic lithotomy, and the hair shaved from the pubic region. A hypodermic of morphine, gr. ¼ Atropia gr. $\frac{1}{150}$ was administered by Dr. Winston, and the anæsthetic of A. C. E. mixture was entrusted to Dr. Pattillo.

So soon as the patient was unconscious the searcher was introduced by Dr. Horsley, who verified his former diagnosis of the existence of several calculi, which was then confirmed by the examination of Dr. Gaston.

An elastic catheter was then introduced into the bladder, and its cavity was washed out with a warm solution of boracic acid, until it returned clear. At this stage a Barnes' Uterine Dilator was rolled lengthwise and passed into the rectum.

A warm solution of boracic acid was injected into the bladder until it contained seven ounces. Eight ounces of warm water being injected into the rubber sack in the rectum, the prominence of the bladder above the pubic bone was detected by the hand over that region. The pelvis was raised by placing a pillow under the patient's hips.

Dr. Horsley now proceeded to make an incision of three inches in length in the median line, extending from the pubic bone upward, and dissected through the adipose tissue and fascia, until the cut was carried in the central part down to the cellular tissue covering the bladder. It was thought best, with a view to lift the peritoneum higher, that the injection should be increased; and as Dr. Horsley had verified on a previous occasion from retention of urine, a capacity of ten ounces for the bladder, the injection of fluid into it was increased to that extent; and the injection in rectal sack was carried to twelve ounces. There was no longer a doubt in regard to the removal of the peritoneum from the field of operation by a finger hooked into the upper angle of the wound, and with a tenaculum securing the walls of the bladder at the lower angle, a cord of four threads of silk was carried by a Peasley needle through the wall of the bladder on each side. These being looped by knotting the ends, were held by assistants, while the final cut into the bladder was made between them to an extent for the finger to be introduced, when the presence of numerous calculi was detected. With the aid of a stone-forceps a calculus corresponding to the size of a partridge egg was immediately extracted, and others of smaller size were removed with the same instrument, when the further progress was interrupted by passing two stitches of iron-dyed silk on each side through the margins of the bladder and skin so as to approximate the cut edges. The boracic solution was now thrown freely as a douche into the bladder, and the search for calculi with the finger was then resumed by Dr. Horsley, when he succeeded in extracting in all twenty-eight calculi of various shapes and sizes.

The warm boracic solution was passed again into the bladder and allowed to run out of the incision until it was free from stain of blood or admixture of any kind.

A very remarkable projection into the bladder was caused by the enlarged prostate gland, and it was found that while there was no difficulty in passing a No. 32 bougie through the urethra, it could not be made to emerge into the cavity of the bladder, owing to the obstacle of the prominent prostate. It was not thought expedient to add to the risks of the patient by attempting any operation upon the prostate on this occasion, and yet at a future day an excision of the middle lobe may be practicable.

The bladder wall was considerably thickened and its stucture rather brittle as was proved by the tenaculum tearing out when a strain came upon it from the escape of fluid by incision, and its friability was also shown by the needle tearing out when first inserted through a small portion of tissue. This would indicate that caution should be observed in not carrying the distention too far by injections, lest rupture might occur into the cavity of the abdomen.

Two drainage tubes, consisting of soft catheters, were secured in the lower angle of the incision by attaching them to the cords left in the walls of the bladder, and condoms were attached to the outer ends to receive the urine. The upper angle of the wound was packed with iodoform gauze, and absorbent cotton was placed over all and a roller passed around the pelvis.

The patient rallied well from the effects of the A. C. E. mixture and had no nausea or vomiting. A notable feature of the case was the request of the old gentleman for a chew of tobacco, which he seemed to enjoy.

In the course of an hour, after returning to consciousness, he complained of some pain and general discomfort, so that he was given another hypodermic of morphia and atropia, which soon quieted him and he went into a quiet sleep. His pulse had been about one hundred before the operation, and continued without alteration two hours afterwards, with a normal temperature.

A letter from Dr. Horsley to Dr. Gaston states, that the patient's condition was excellent, so far as the operation was concerned, on the subsequent day. At 7 A. M., pulse, 100; temperature 100°; respiration, 20. 2 P. M., pulse, 84; temperature, 100°; respiration, 20. Resting well.

The urine escapes clear through wound and drainage tubes. The bladder was washed out with boracic solution during the morning.

Dr. Huzza, in expressing his appreciation of Dr. Gaston's report, referred to position of patient during operation. Said by placing patient's head on a lower level than his body, gravity would remove all danger to peritoneum. Dr. Elkin said, the peritoneum could readily be pushed out of way by the hand of the operator. Said further, that he thought the suprapubic operation for case in hand, preferable to any of the perineal operations.

A letter was received by Dr. Gaston on March 24th, in which Dr. Horsley says: "Our patient continues to improve steadily, wound looks healthy, appetite good and sleeps well. Removed the stitches Friday evening, 21st inst., and the drainage tube yesterday; patient has very little pain since. I now consider the patient as being safe from the effects of the operation performed a week ago, and hope that the drainage will secure him relief to the cystitis."

NOTE.—The following statements from Dr. Horsley give additional interest to the above report of the case:

"Lee James, white, æt. 76 years; saw case first on October

27th, 1889. Patient gave history of having suffered from bladder trouble for three years; had been under care of various physicians, some of whom had examined for calculus, but without success. Examination of urine showed it loaded with crystals of triple-phosphate and uric acid. One week later an exploration with Thomson's sound revealed the presence of a stone apparently about one-half inch in diameter. On November 20th, 1889, after some preparatory treatment an attempt was made, with the assistance of Drs. Patillo and Winston, to crush the stone with Civiale's instrument. The stone could not be grasped, although the patient was etherized. On the 28th of November, however, the patient being etherized by Dr. Patillo, Dr. Winston acting as assistant, litholapaxy was successfully performed, and two stones, one-half and one-fourth inch in diameter, were crushed and were evacuated with Otis' apparatus. No more calculi could be felt at the time.

Patient bore all the manipulations well, but continued to suffer with the evidences of calculus. Examinations revealed, though imperfectly, the presence of calculi, but attempts with Civiale's and Bigelow's instruments failed to catch stone. Patient's sufferings increasing, suprapubic cystotomy was decided upon."

Dr. Huzza presented the ovaries from a case of Battey's operation, which he will report in full at another meeting.

"Mrs. H., æt. 35, widow, was thrown from a horse thirteen years ago; after a long illness partially recovered, but had a tumor to appear in the right side. Began to suffer with violent dysmenorrhœa; instead of yielding to treatment it gradually grew worse, till now she takes large quantities of morphine to relieve the constant pain, which is greatly exacerbated every month. Has not been out of her room for three years. Was under Dr. Thomas, of New York, six years ago, who advised an operation, but she refused. For the last year has been under care of Dr. J. C. Avary, who called me in consultation. We operated March 18th, 1890. Right ovary, cystic, size of duck egg; left ovary, cirrhotic, shrunken, adherent. Tubes showed patches of lymph and small peritoneal cyst. Both ovaries and tubes were entirely removed. Patient is rapidly convalescing."

Dr. Stockton reported a case of fish bone in the larynx. Mr. D. O., æt. 30, came to me February 25th; said he had a bone in his throat, and that he had already been to see two physicians, and that they had put instruments and their fingers down his throat until he could stand it no longer. On examination, throat was found raw and very much inflamed, so that it was impossible to examine it in that condition. I used a spray of cocaine, 10 per cent., and after waiting five minutes, used the laryngoscope, and at once detected the bone running laterally, and place above the true cords and below the false. I had no difficulty in extracting the bone at the first attempt by means of MacKenzie's antero-posterior forceps.

In connection with report of fish bone in the *fauces*, reported by Dr. Stockton, it was stated by Dr. Gaston that some years ago he encountered a pin lodged in such a manner that the point penetrated the base of epiglottis, while the head was embedded in the tonsil. He seized the pin with the gullet forceps, and releasing one extremity by pressure of the index finger, the pin was extracted.

Dr. G. H. Noble reported a case where prolapse of ovaries gave rise to a state of catalepsy.

Dr. Virgil O. Hardon reported a case of superficial papilloma of both ovaries, presenting specimens.

Dr. M. B. Hutchins wished, in fairness, to report that the case of acne of the back, mentioned as doing well under ½ to 1 per cent. bichloride solution and soap frictions, had since got worse, and was now improving under the lotio alba cum. sulph. (℞ Zinci Sulph.; Potass. Sulphuret āā, ʒi; Aquæ, ℥iv. M. after separate solution of ingredients each in ℥ii. of the water. Then add sulphur ʒi), which is the standard acne lotion in the New York Skin and Cancer Hospital. Strength of lotion is varied according to indications. Good effect probably due to the sulphur elements.

At the request of Dr. Baird, the treatment of acne, as affecting the face of young ladies, was gone into quite fully.

Dr. H. P. Cooper in discussion, said the subject of skin diseases had always been of special interest to him. He was once pretty familiar with the different skin lesions and their appropriate names, but he had forgotten them, and that he now tried to exclude syphilis and the parasitic diseases from the case, and then divided his treatment into sedative and stimulative, according as the disease was acute or chronic.

Dr. Hutchins replied by citing a case seen that morning which suggested syphilis, ring-worm, psoriasis and *eczema seborrhoicum*. Diagnosed the latter, though at first almost decided upon psoriasis. This case, treated on general principles, would not recover. Mentioned a case which a specialist in New York treated twelve months for psoriasis. Got well in six weeks after treatment for seborrhoic eczema, which it proved to be. Yet appearances seemed the same. L. P. K.

